# Suppressing Anaphase-Promoting Complex/Cyclosome–Cell Division Cycle 20 Activity to Enhance the Effectiveness of Anti-Cancer Drugs That Induce Multipolar Mitotic Spindles

**DOI:** 10.3390/ijms25126329

**Published:** 2024-06-07

**Authors:** Scott C. Schuyler, Hsin-Yu Chen, Kai-Ping Chang

**Affiliations:** 1 Department of Biomedical Sciences, College of Medicine, Chang Gung University, Kwei-Shan, Taoyuan 333, Taiwan; 2 Department of Otolaryngology—Head and Neck Surgery, Chang Gung Memorial Hospital, Taoyuan 333, Taiwan; 3 Molecular Medicine Research Center, Chang Gung University, Taoyuan 333, Taiwan

**Keywords:** cancer, anaphase-promoting complex/cyclosome (APC/C), cell division cycle 20 (CDC20), paclitaxel

## Abstract

Paclitaxel induces multipolar spindles at clinically relevant doses but does not substantially increase mitotic indices. Paclitaxel’s anti-cancer effects are hypothesized to occur by promoting chromosome mis-segregation on multipolar spindles leading to apoptosis, necrosis and cyclic-GMP-AMP Synthase–Stimulator of Interferon Genes (cGAS-STING) pathway activation in daughter cells, leading to secretion of type I interferon (IFN) and immunogenic cell death. Eribulin and vinorelbine have also been reported to cause increases in multipolar spindles in cancer cells. Recently, suppression of Anaphase-Promoting Complex/Cyclosome–Cell Division Cycle 20 (APC/C-CDC20) activity using CRISPR/Cas9 mutagenesis has been reported to increase sensitivity to Kinesin Family 18a (KIF18a) inhibition, which functions to suppress multipolar mitotic spindles in cancer cells. We propose that a way to enhance the effectiveness of anti-cancer agents that increase multipolar spindles is by suppressing the APC/C-CDC20 to delay, but not block, anaphase entry. Delaying anaphase entry in genomically unstable cells may enhance multipolar spindle-induced cell death. In genomically stable healthy human cells, delayed anaphase entry may suppress the level of multipolar spindles induced by anti-cancer drugs and lower mitotic cytotoxicity. We outline specific combinations of molecules to investigate that may achieve the goal of enhancing the effectiveness of anti-cancer agents.

## 1. Introduction

Cancers have been the leading cause of death in Taiwan and Japan for the past 41 and 43 years, respectively, and currently are responsible for about 1/6th of deaths globally [1,2,3]. The advent of novel anti-mitotic, anti-cancer drugs is thought to have been impeded by mitotic cytotoxicity in healthy cells, leading to many failed clinical trials [4,5]. Molecules that promote the cell death of cancer cells by targeting cell division are hypothesized to induce mitotic cytotoxicity in healthy populations of rapidly dividing cells in adults, leading to the development of, among other side effects, neutropenia, alopecia and mucositis, limiting the dose at which the drugs can be used in the clinic, and thus limiting their effectiveness [5,6,7]. The high levels of mitotic cytotoxicity within healthy dividing cells may reflect the fact that a drug designed to target a protein that contributes to mitosis will disrupt the target’s function equally in healthy cells and in cancer cells during mitotic progression. This hypothesis, combined with the fact that there are cellular populations in the adult body that are thought to divide much faster than the average tumor or cancer cells [5], has led to the conclusion that it may be necessary to focus only on mitotic targets that display some established differential between healthy cells and cancer cells, potentially making the cancer cells more vulnerable to the chemotherapeutic agent. Currently, one of the best potential examples in the development of this kind of anti-mitotic cancer cell-specific agent are molecules such as the sovilnesib (AMG 650)-targeting Kinesin Family 18a (KIF18a), which, in normal cells, is non-essential in mitosis but in some cancers displays an essential phenotype when depleted, with KIF18a inhibitors potentially increasing the frequency of multipolar spindles in cancer cells (clinical trial: NCT04293094) [8].

In addition to concepts focused on trying to select vulnerable cancer cell-specific targets, the success of paclitaxel (Taxol) as a chemotherapeutic agent has been a lighthouse guiding thought on the development of novel anti-cancer drugs, along with other related agents, such as docetaxel. Paclitaxel is anti-mitotic in action, but the source of its therapeutic index, namely the difference between its dose-dependent ability to promote tumor regression compared to its cytotoxic side effects, remains unknown. It has been hypothesized that one source of paclitaxel’s therapeutic index may be the induction of leukocyte recruitment into solid tumors causing immunogenic cell death and tumor regression [4,5,6,7,9,10]. White blood cell recruitment depends upon cytokine signaling via a class of chemokine interferons, with one example being type I interferon-b (IFN-b) [11]. The initiation of the production of IFN-b depends upon the activation of the cGAS-STING pathway, which is activated in response to the presence of exposed single-strand and double-stranded DNA in the cytosol [12,13].

Thus, the current hypothesis is that the therapeutic index of 10 nM paclitaxel, the clinically relevant amount to expose MDA-MB-231 breast cancer cell lines to in vitro [14], does not involve substantial cell-cycle arrest in mitosis, but rather may be derived from the formation of multipolar mitotic spindles that can lead to two different cell death fates after a mother cell with a multipolar spindle executes an aberrant mitosis, namely (i) an apoptosis/necrosis/quiescence pathway and (ii) an immunogenic cell death pathway [4,5,9,10,14,15,16]. Eribulin and vinorelbine, two other anti-cancer, anti-mitotic agents, like paclitaxel, were recently observed to promote multipolar spindles in situ in tumor biopsy samples and in vitro in tissue culture cells, even though they induce microtubule depolymerization instead of microtubule stability like paclitaxel [16]. The implications of the results of these clinical trials [9,14,15,16] and cell-line studies on paclitaxel, eribulin and vinorelbine are profound: post-treatment in situ tumor cell analyses revealed only small increases in mitotic indices (5% or less) and large increases in the number of multipolar mitotic spindles (between 10 and 60%) in the cancer cells. For paclitaxel, these levels correspond to 10 nM paclitaxel in MDA-MB-231 and CAL-51 breast cancer cell lines, with a limited increase in mitotic index (20% or less) as the key factor which establishes this limit in the mitotic index as an experimental cut-off for clinical relevancy. Namely, in any given cancer-model cell line, the molar limit of paclitaxel for clinical relevancy may be a mitotic index of 20% or less. This is a radical change in thinking: the purpose of an anti-mitotic agent like paclitaxel is not to induce a mitotic block or arrest but rather to induce chromosome mis-segregation events on multipolar mitotic spindles in a manner that allows the mitotic spindle checkpoint to be satisfied, thus avoiding both a mitotic block and cell death in mitosis and potentially decreasing the risk that the anti-mitotic agent induces mitotic cytotoxicity in healthy human cells, leading to adverse conditions, such as neutropenia, at the clinically relevant dose. 

In eukaryotic mitosis, the metaphase–anaphase transition is controlled by the APC/C, an E3-ubiquitin ligase; and its essential co-factor, CDC20 [17,18]. As cells enter prophase, the APC/C-CDC20 enzyme remains inactive due, in part, to the activity of the mitotic spindle assembly checkpoint [19,20]. When replicated paired-sister chromatids are not properly attached and placed under tension by microtubules of the mitotic spindle, they generate the formation of the mitotic checkpoint complex (MCC), which contains MAD2, the Mitotic Arrest Deficient 3 (MAD3/BUBR1)-Budding Uninhibited by Benomyl 3 (BUB3) complex and CDC20, and the formation of this complex blocks the activation of APC/C-CDC20 arresting cells in prometaphase/metaphase [19,20]. Mitotic checkpoint regulation of the metaphase–anaphase transition is essential for accurate chromosome segregation and viability in mammals [19,20], and reagents that target the activity of APC/C-CDC20 have been suggested to have potential value as a way to target and control mitotic progression in cancer cells [21,22,23,24,25]. In a groundbreaking result, it has recently been reported that KIF18a inhibition by the small molecule AM-1882 was enhanced by lowering APC/C-CDC20 activity by CRISPR/Cas9 mutagenesis and validated via an analysis of the RNAi results [26]. This creates the possibility that specifically lowering APC/C-CDC20 activity may be employed as a strategy to augment established anti-cancer agents that promote/increase multipolar mitotic spindles like paclitaxel, eribulin and vinorelbine, or augment a potential anti-cancer agent currently under development, like sovilnesib (AMG 650), which inhibits KIF18a.

## 2. Paclitaxel, Eribulin and Vinorelbine Are Hypothesized to Act via Mitotic Cytotoxicity by Inducing Multipolar Spindles, Leading to Daughter Cell Death

**Figure 1:**
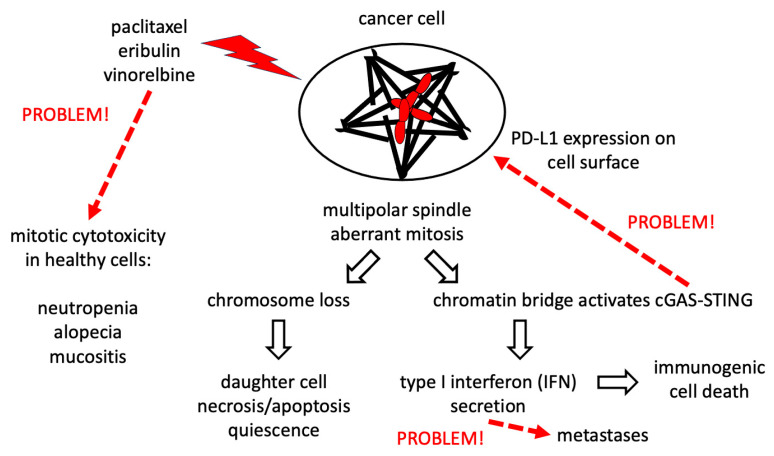
The therapeutic index of 10 nM paclitaxel is hypothesized not to involve cell-cycle arrest in mitosis but may be derived from the formation of multipolar mitotic spindles in genomically unstable cancer cells. Multipolar mitotic spindles are proposed to lead to two different cell death fates after a mother cell with a multipolar spindle executes an aberrant mitosis, namely daughter cell death/arrest (left pathway) and immunogenic cell death (right pathway).

Paclitaxel is approved in the USA for the treatment of more than 20 types of adult cancers [27]. The therapeutic index of 10 nM paclitaxel, the clinically relevant amount to work with in lab-cultured MDA-MB-231 cell lines [14], is now hypothesized not to involve cell-cycle arrest in mitosis, but rather may be derived from the formation of multipolar mitotic spindles that can lead to two different cell death fates after a mother cell with a multipolar spindle executes an aberrant mitosis [4,5,9,10,14,15,16] (Figure 1). 

Elevated levels of chromosome mis-segregation on the multipolar spindle may induce necrosis/apoptosis/quiescence in the daughter cells due to a loss of genetic information and/or cell stress induced by imbalances in transcriptional networks, resulting in dysfunctional and proteo-toxic protein expression levels [9,14,15,16]. Second, a lagging chromosome(s) in anaphase on the multipolar spindle of the mother cell may lead to a chromatin bridge(s) in cytokinesis that activates the cGAS-STING pathway in the daughter cells, which may promote type I interferon secretion, resulting in leukocyte recruitment into solid tumors which execute immunogenic cell death, a pathway that can also be activated during apoptosis and/or necrosis [4,5,9,10,28,29]. The efficacy of paclitaxel has been enhanced by combinatorial therapies based on anti-immune checkpoint drugs targeting programmed death-ligand 1 (PD-L1)/programmed cell death protein 1 (PD-1) (for example, see essay on head and neck cancers [30]). Many patients do not respond to paclitaxel treatments before critical limiting doses of paclitaxel are reached, caused by adverse side effects such as neutropenia, mucositis and/or neurotoxicity [4].

We wish to emphasize that this hypothesis, which centers on daughter cell death, as the basis for the therapeutic index of paclitaxel was only developed in about the last 10 years, and that it is not at all universally accepted by any means. Overwhelmingly, work with paclitaxel for the past 5+ decades exposed cells to amounts that are much higher than 10 nM, leading to alternative results. Namely, at such high doses, paclitaxel induces a sustained, long-duration mitotic block, resulting in the execution of apoptosis and/or necrosis in mitosis [14]. This is a mother cell mitotic death event, not a daughter cell death event(s). To our knowledge, the clinically relevant dose has only been clearly defined in the breast cancer models of MDA-MB-231 cells and CAL-51 cells [14]. The establishment of the clinically relevant dose required the following measurements: (i) a measurement of the cellular amount of paclitaxel from clinical biopsy tumor samples isolated from breast cancer patients who had just received their chemotherapy dose, and (ii) an intracellular measurement of the amount of paclitaxel after a treatment of breast cancer cell model-system cells in vitro. A complicating feature of paclitaxel is that cells act as ‘molecular sponges’ by absorbing a large amount of paclitaxel from their surroundings so that the intracellular levels reach micromolar amounts even when the cells are exposed only to nanomolar amounts [14]. This ‘molecular sponge’ effect is thought to have its basis in the fact that paclitaxel’s targets in the cell are the microtubules, which are highly abundant. Although there have been other inter-tumor and intracellular measurements of paclitaxel, they have not been combined together with matched cell lines and matched biopsy samples in a manner that allows for a clear statement of what the proper clinically relevant dose might be for a given cancer-model cell line. Furthermore, to make matters more confounding, to our knowledge, the clinically relevant doses for eribulin and vinorelbine have also not yet been established in this compelling way for in vitro cell culture-based experimentation. However, progress in the right direction is being made [16].

Like paclitaxel, eribulin and vinorelbine were recently reported to induce the formation of multipolar spindles in cell lines in vitro and in tumor cells isolated in cancer biopsy samples shortly after treatment in situ [16]. This breakthrough report indicates that the therapeutic indexes of these three drugs may have the same cellular origin, namely anaphase entry in the presence of multipolar spindles. Although these anti-cancer agents share this similarity, fewer details are known about the consequences of exposing cancer cells to eribulin or vinorelbine as compared to paclitaxel at the clinically relevant doses, especially regarding the potential promotion of immunogenic cell death. However, even within these more limited sets of data, there are intriguing common features among the bioactivity between these three anti-cancer agents. 

Eribulin is known to cause a decrease in the normal level of chromosome oscillations in metaphase, a condition that may decrease accurate chromosome segregation [31] (Figure 1). Eribulin can also cause alopecia and neutropenia, which may reflect its level of inducing mitotic cytotoxicity in rapidly dividing healthy human cells [32]. But, to our knowledge, a direct measurement of the levels of chromosome mis-segregation induced by eribulin has not yet been made. Exposure to eribulin also induces apoptosis, but we are not aware of data showing a correlation between activated regulated cell death and the quantitative scale of chromosome mis-segregation events [33]. It is also not known if eribulin increases the frequency of the formation of chromatin bridges; however, it has been observed that eribulin does increase the frequency of micronuclei, an indication of elevated levels of chromosome mis-segregation, and these micronuclei have been observed to be cGAS-positive, indicating the potential activation of the cGAS-STING pathway [10]. However, it has also been observed that cGAS-STING activation by eribulin depends on mitochondrial DNA [34]. Nonetheless, eribulin can promote the production of IFN-b, a type-I interferon, and promote immunogenic cell death [35]. As with paclitaxel, eribulin treatment can lead to the elevated expression of PD-L1, and, in combination with interferon secretion, leads to concerns about the suppression of immunogenic cell death and an enhancement of metastases (Figure 1) [28].

Vinorelbine promotes multipolar spindles in tissue culture cells and in breast cancer biopsy samples and is also known to induce neutropenia (Figure 1) [36]. The segregation of chromosomes on multipolar spindles has been observed to cause elevated levels of chromosome mis-segregation events that induce daughter cell death [16]. Vinorelbine also leads to the activation of the cGAS-STING pathway and production of type I interferons, at the least creating the possibility for the promotion of immunogenic cell death, although we are not aware of experimental evidence to support this speculation [37]. Type I interferon secretion also raises concerns about metastases. Like paclitaxel and eribulin, vinorelbine activates the expression of PD-L1 on the surface of cancer cells, leading to concerns about the suppression of immunogenic cell death.

## 3. Suppressing APC/C-CDC20 Sensitizes Cells to KIF18a Inhibition

The inhibition of KIF18a, typically by an RNAi knockdown-based method, and more recently by small-molecule inhibition using AM-1882 or sovilnesib (AMG 650), is known to suppress the ability of some cancer cells to decrease the levels of multipolar mitotic spindles, establishing a similarity with the mechanism of action of paclitaxel. In a recent breakthrough paper, Gliech and co-workers performed a CRISPR/Cas9 knockout screen to identify proteins that could sensitize cells to the KIF18a inhibitor AM-1882 in cells that had been selected to be polyploid, which may have sensitized the cells to become genomically unstable, in part, because they may have multiple microtubule-organizing centers which would imply the formation of multipolar spindles in mitosis [26]. They identified several subunits of the APC/C, including APC4 and APC5, and also found a co-dependency between KIF18a function and APC1, APC4, APC8 and APC3 by further RNAi data analyses [26]. This creates the possibility that delayed anaphase entry may enhance the lethality of paclitaxel in genomically unstable cancer cells via a mechanism similar to that proposed for KIF18a inhibition. However, whether KIF18a inhibition has therapeutic value remains unknown, as the results of an initial and a second current clinical trial on sovilnesib (AMG 650) have not been reported (clinical trial: NCT04293094).

## 4. Suppressing APC/C-CDC20 May Enhance the Effectiveness of Anti-Mitotic Chemotherapeutic Drugs That Induce Multipolar Spindles without Substantially Increasing the Mitotic Index

**Figure 2:**
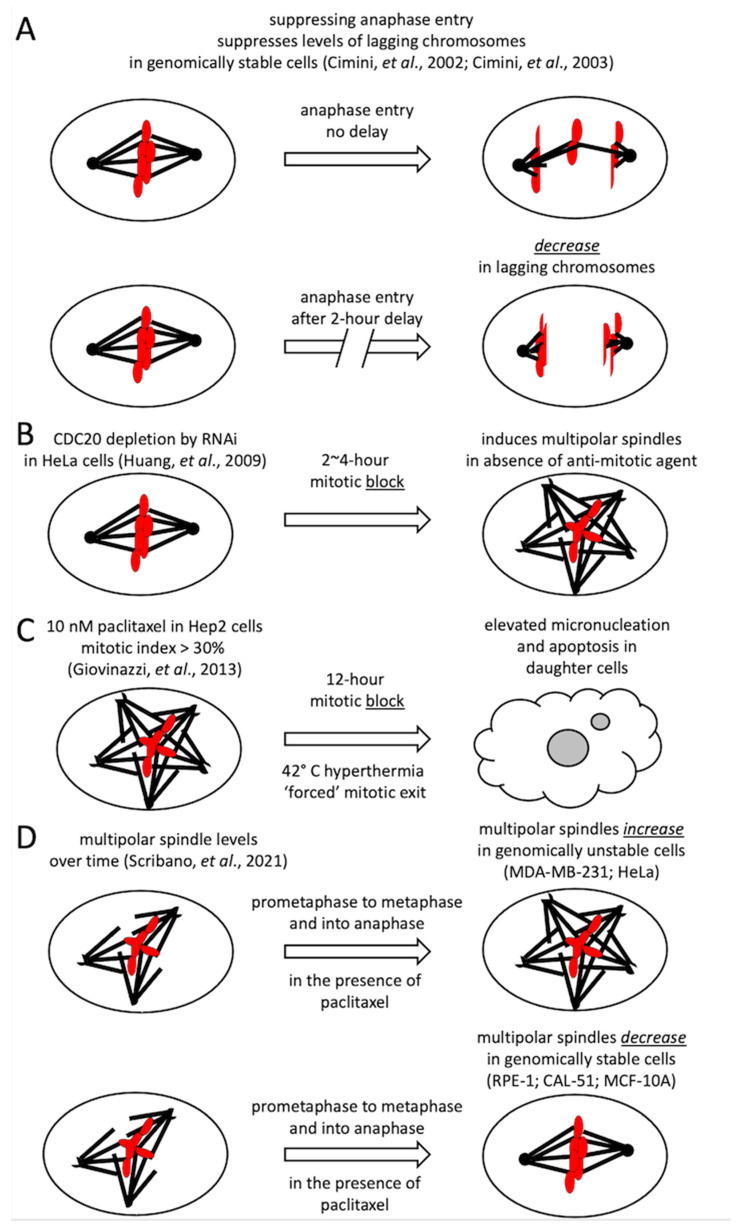
The critical observations from five previous studies. (**A**) Genomically stable cells may decrease chromosome mis-segregation in response to delayed anaphase entry [40,41]. (**B**) Genomically unstable cancer cells my increase the formation of multipolar spindles [42] and (**C**) chromosome mis-segregation in the presence of paclitaxel in response to delayed anaphase entry [43]. (**D**) These results are consistent with the observations that genomically unstable cancer cells (MDA-MB-231 and HeLa) tend to increase the levels of multipolar mitotic spindles over time in the presence of paclitaxel, while genomically stable cells (RPE-1, CAL-51, and MCF-10A) tend to decrease the levels of multipolar mitotic spindles over time in the presence of paclitaxel [9].

To potentially enhance anti-cancer agents that induce or increase the levels of multipolar spindles, such as paclitaxel, eribulin, vinorelbine or sovilnesib (AMG 650), we propose to specifically target the Anaphase-Promoting Complex/Cyclosome–Cell Division Cycle 20 (APC/C-CDC20) enzyme complex to try to create a temporary *delay* in anaphase entry without inducing a mitotic cell-cycle arrest, or an extended mitotic *block* [38,39]. The basis of this hypothesis largely stems from five previous reports [9,40,41,42,43]. First, Cimini and co-workers observed that a 2-h temporary delay in anaphase entry induced by the 26S proteosome inhibitor MG-132 in genomically stable cells with or without the prior treatment of the spindle-damaging agent nocodazole suppressed the number of lagging chromosomes (Figure 2A) [40,41]. These experiments revealed an error-correction mechanism that could function over time to suppress chromosome mis-segregation errors induced by mitotic poisons. These observations indicated that, in healthy cells, a 2-h delay in metaphase may suppress the adverse effects of anti-mitotic drugs in a genomically stable cell. Then, in a foundational paper, Huang and co-workers observed that a 2~4-h block in metaphase could induce spindle pole fragmentation in genomically unstable HeLa cancer cells (Figure 2B) [42]. Third, Hep2 larynx cancer cells arrested in a paclitaxel-induced mitotic block with a high mitotic index of over 30% could be forced to exit mitosis via hyperthermia, resulting in daughter cells with observed elevated levels of micronuclei and increased levels of apoptosis (Figure 2C) [43]. The presence of micronuclei strongly suggests increased levels of chromosome mis-segregation. Hyperthermia is clinically employed in cancer therapy [43]. This observation showed that mitotic progression, even forced mitotic progression, in a cancer cell in the presence of a multipolar mitotic spindle could increase rates of chromosome mis-segregation and lead to elevated rates of cancer cell death.

Finally, based on the observations of Scribano et al. (2021) and using paclitaxel as the example, we speculate that delaying anaphase entry in the presence of 10 nM of paclitaxel may decrease multipolar spindles in genomically stable healthy cells and increase multipolar spindle formation in genomically unstable cancer cells [9]. Levels of multipolar spindles induced by 10 nM paclitaxel have been observed to decrease from prophase to metaphase in genomically stable CAL-51 cells, and they have also been observed to decrease from metaphase into anaphase in genomically stable CAL-51, RPE-1 and MCF-10A cells, while, in contrast, in genomically unstable cells, like MDA-MB-231 and HeLa cells, the levels of multipolar spindles have been observed to increase from metaphase into anaphase (Figure 2D) [9]. Healthy human cells have been selected to be genomically stable and are very rarely aneuploid [44,45], while cancer cells have been selected to be genomically unstable, frequently resulting in aneuploidy [46]. Thus, in the presence of 10 nM paclitaxel, a delay in anaphase entry may give genomically stable cells more time to repair their multipolar spindles to spindles that are bipolar in form, while genomically unstable cells delayed in anaphase entry may display an increase in multipolar spindles in anaphase, leading to increased levels of chromosome mis-segregation events and cancer cell death. 

Delayed anaphase entry in the presence of anti-cancer, anti-mitotic agents also enhances the activation of the ‘mitotic stopwatch’ pathway, involving p53, p21, p53-binding protein 1 (53BP1) and ubiquitin-specific protease 28 (USP28). This pathway leads to increased levels of daughter cell arrest and the promotion of daughter cell death in cancer cells that remain sensitive to this pathway [47,48,49,50,51,52]. The question now becomes: are there any anti-anaphase entry agents that can be investigated in combination with anti-mitotic agents such as paclitaxel, eribulin, vinorelbine and sovilnesib (AMG 650)?

**Figure 3:**
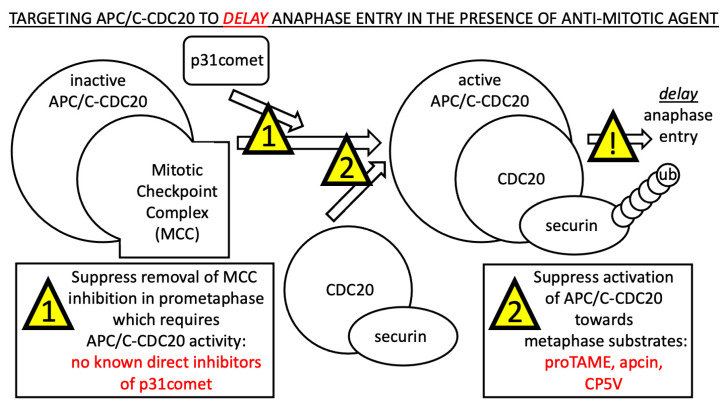
Proposed steps to target to *delay* anaphase entry during the sequential activation of APC/C-CDC20 from prometaphase to metaphase in the presence of the clinically relevant dose of an anti-mitotic agent such as paclitaxel. The most promising current candidates are proTAME, apcin and CP5V.

The current challenge to work towards potentially more effective anti-cancer combinatorial treatments that can be widely available and affordable is to test this hypothesis directly through experimentation. There are several cell-permeable pre-clinical molecules that have been discovered and/or designed to target APC/C-CDC20, with proTAME, apcin and CP5V perhaps being the best characterized molecules that interact directly with the APC/C-CDC20 to inhibit activity (Figure 3). In prometaphase, APC/C-CDC20 remains inactive towards many, but not all, target substrates because the mitotic spindle checkpoint remains ‘unsatisfied’. Once the mitotic spindle checkpoint is ‘satisfied’ by all chromosomes being properly attached to and placed under tension by the mitotic spindle, p31comet continues to participate in the removal of the MCC from the APC/C-CDC20 holo-enzyme complex, which is proposed to require APC/C activity itself (step 1) [53]. Next, after MCC is removed, the free and activated form of the APC/C is proposed to associate with a CDC20 co-factor/substrate complex, such as securin, to catalyze the poly-ubiquitinylation (ub) of the target substrate (step 2). We speculate that suppressing the removal of MCC inhibition in prometaphase (step 1) and/or suppressing APC/C-CDC20 activation towards metaphase substrates (step 2) may yield delayed anaphase entry. It is critical to note that we are proposing to only *delay* anaphase entry, and not to *block* anaphase entry via a cell-cycle arrest or via an extended mitotic checkpoint-dependent arrest, out of concern that blocking the cell cycle in prometaphase/metaphase may cause mitotic cytotoxicity in healthy human cells in both the presence and absence of anti-cancer mitotic poisons. In order to achieve the goal of delaying anaphase entry by targeting the APC/C-CDC20, the most promising current candidate molecules are proTAME, apcin and CP5V.

## 5. Discussion

**Figure 4:**
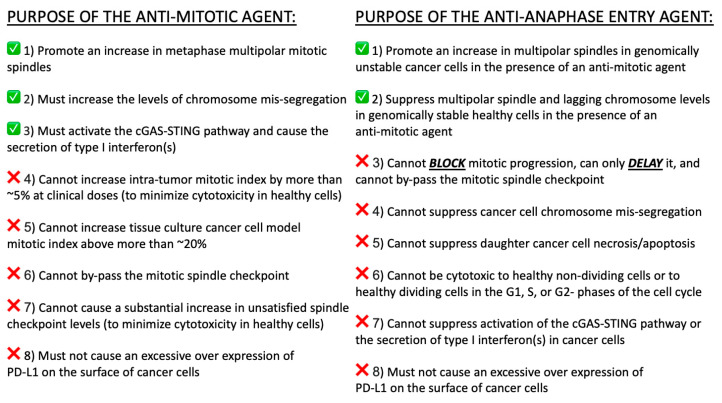
Proposed lists of criteria for interpreting the purpose of anti-mitotic, anti-cancer agents and a corresponding list of criteria for considering the purpose of anti-anaphase entry agents. The goal of performing experiments moving forward is to measure each one of these criteria by studying anti-anaphase entry molecules. The hope is that agents will be identified that have the potential to suppress mitotic cytotoxicity in healthy human cells and enhance mitotic cytotoxicity in cancer cells.

When evaluating molecules that target APC/C-CDC20 to try to enhance the anti-cancer activity of established anti-mitotic agents that induce/increase multipolar spindles, a list of several criteria should be noted, which are based upon current knowledge and understanding of their mechanisms of action (Figure 4).

The small molecule proTAME transits the plasma membrane and is activated by esterase-dependent cleavage inside of cells and then targets the APC/C co-factor Isoleucine–Arginine (IR)-tail binding site on the Anaphase Promoting Complex 3 (APC3) subunit [21,22]. Once activated in the cell by cleavage, the TAME form of the molecule can no longer efficiently cross the plasma membrane, making it an irreversible inhibitor at the level of the cell [21,22]. The active cellular TAME molecule inhibits both the CDC20 and CDC20-like Homolog 1 (CDH1) forms of the APC/C, thus not only disrupting APC/C function in mitosis but also in other portions of the cell cycle, such as the G1 phase of the cell cycle, where APC/C-CDH1 activity is essential, as well as potentially disrupting essential neurological functions of the APC/C [21,22]. However, suppressing APC/C-CDH1 activity might have the benefit of promoting the formation of multipolar mitotic spindles [54]. Direct tests will have to be performed to investigate the combined effects at the clinically relevant dose of the anti-mitotic agent and at a low non-cytotoxic dose of proTAME. There have also been studies in Hep2 cells on combined exposure to paclitaxel and proTAME, but the levels of the resultant mitotic index were too high (mitotic index > 30%), and the levels of proTAME employed (12 mM) were also quite high [43].

The small organic molecule apcin is an effective inhibitor specifically of the APC/C-CDC20 form of the APC/C by targeting the CDC20 co-factor, which gives it the potential advantage that it might have more limited side effects in vivo because the only known essential function of CDC20 in adults is the execution of the metaphase–anaphase transition [22,24,25]. Although the APC/C plays critical roles in non-dividing cells, we are not aware of any essential function for the APC/C-CDC20 form of the APC/C beyond its role in cell division in adults. But apcin also has an unanticipated effect on the ability of the MCC to function in vivo simultaneously disrupting APC/C-CDC20 activation and MCC-dependent APC/C-CDC20 inhibition in the same cell, which promotes partial cell cycle progression into anaphase even in the presence of a mitotic poison [24]. 

The PROteolysis TArgeting Chimera (PROTAC) molecule CP5V is a thalidomide-linked form of apcin that is designed to bind with the von Hippel-Lindau (VHL) E3-ligase and CDC20 to promote the poly-ubiquitinylation and proteolytic destruction of CDC20 protein, blocking mitotic progression in metaphase, which leads to apoptosis after an extended metaphase arrest [23]. In fact, all three of these molecules have been observed to promote mitotic cytotoxicity, which may have valuable anti-cancer effects [21,22,23,24,25]. However, the value of inducing general non-cancer cell-specific mitotic cytotoxicity as an anti-cancer strategy has been strongly challenged because of concerns about the effects of mitotic cytotoxicity on other rapidly dividing healthy cells in the adult human body, such as promyelocytes (see, for example, [4,5,16]).

The APC/C has been established to have auto-inhibitory loops that are regulated by cell cycle-dependent phosphorylation [18,55]. Thus, taking a cue from nature, in addition to these small molecules, artificial peptides have been observed to inhibit APC/C-Cdc20 activity in vitro that can lead to delayed anaphase entry in vivo when the peptide was overexpressed [38]. The yeast peptide ‘DQ36’ (197-DMNKRILQYMPEPPKCSSLRQKSYIMKKRTHYSYQQ-132), derived from the N-terminus of the budding yeast Cdc20 co-factor protein, was observed to block APC/C-Cdc20 activity in vitro by binding directly to the APC/C and suppressing the association of the Cdc20 co-factor with the holo-enzyme complex, bioactivities that were displayed by several other peptides too [38] (see Figure 8 in [38]). The potential human cognate peptide of yeast DQ36 may be the human (124-DVEEAKILRLSGKPQNAPEGYGNRLKVLYSGKATPG-159) ‘DG36’ peptide, where the underlined portion of the peptide represents the portion of the molecule that has been resolved structurally and contains the ‘KILR’-motif that targets the APC8B subunit [18,56]. Further tests on these types of peptides [38] and ones from a similar region in human CDC20, or their potentially more stable retro-inverso analogues, merits investigation in human cancer cells.

Finally, as noted earlier, cancers have been the leading cause of death in Taiwan for the past 41 years; currently, they are responsible for about 1/6th of deaths globally, and the affordability of treatment remains a major issue [1,2,3]. In Taiwan, which is considered to have a world-class health-care system, for example, the combination of platinum–paclitaxel–anti-PD-l treatment, which is suggested to be the best current approach targeting head and neck cancers [30], is a new direction of therapy for recurrent advanced/metastatic head and neck squamous cell carcinoma. However, the toxicity is higher, and patients may need to pay for paclitaxel or anti-PD-1 therapies out of their own pocket. Anti-PD-1 antibodies, either pembrolizumab or nivolumab, are reimbursed by the national health insurance only under certain indications, such as tumor proportion score (TPS) > 50% or combined positive score (CPS), combining all scores (or proportions) of PD-L1 positive tumor cells, tumor-infiltrating lymphocytes (TILs) and macrophages in the immunohistochemical staining of patient tumor tissues, CPS > 10%, respectively. Currently, the reality is that these financial obstacles limit the utility of this recommended approach in clinical practice in Taiwan even in the context of an advanced health-care system. 

## 6. Conclusions

We proposed that suppressing the APC/C-CDC20 may be a way to delay anaphase entry in mitosis to specifically enhance the anti-cancer effects of a class of established and potentially novel chemotherapeutic agents that are hypothesized to execute their anti-cancer cell effects by promoting the formation of multipolar mitotic spindles during cell division and also have the potential to activate the mitotic stopwatch pathway [47,48,49,50,51,52]. This approach can be tested by employing the FDA-approved anti-cancer drugs paclitaxel, eribulin and vinorelbine, as well as by testing experimental KIF18a inhibitors such as sovilnesib (AMG 650) that are currently undergoing clinical trials. These anti-mitotic agents can be tested in combination with current known small-molecule inhibitors of the APC/C, such as proTAME, apcin and CP5V, at non-cytotoxic low doses to investigate if they display a combined effect [21,22,23,24,25]. In addition, the development of other novel molecules, such as those based on peptide inhibitors of the APC/C-Cdc20 in yeast, should be investigated [38]. These combinatorial treatments will need to be explored in a variety of cancer cell-line models to evaluate their potential utility to combat against the broad spectrum of cellular diversity of cancer types that are encountered in the clinic. The hypothesis outlined here, that a *delay* in anaphase entry, but not a *block* in anaphase entry, can suppress genomic instability in healthy cells and enhance genomic instability in cancer cells in the presence of the clinically relevant doses of anti-cancer agents that promote the formation of multipolar mitotic spindles remains to be tested but is both plausible and experimentally within reach.

## 7. Materials and Methods

The National Center for Biotechnology Information (NCBI) (https://www.ncbi.nlm.nih.gov/, accessed on 15 March 2024) database was used to obtain human protein sequences, and the Saccharomyces Genome Database (SGD) (http://www.yeastgenome.org/, accessed on 15 March 2024) was used to obtain yeast protein sequences [57,58,59]. The basic local alignment search tool (BLAST) tool at NCBI was used for initial amino acid-sequence alignments [57,58,59]. Cryo-electron microscopy structures were analyzed using the Research Collaboratory for Structural Bioinformatics Protein Data Bank (RCSB PDB) (https://www.rcsb.org/, accessed on 15 March 2024) database [60,61].
